# Amyotrophic lateral sclerosis -plus patient with an intermediate-length *CACNA1A* allele: a Case Report

**DOI:** 10.3389/fgene.2026.1860828

**Published:** 2026-08-19

**Authors:** Xinyao Gao, Tingting Wang, Sihui Chen, Yuanzheng Ma, Qianqian Wei, Chunyu Li, Huifang Shang, Xueping Chen

**Affiliations:** 1 Department of Neurology, West China Hospital, Sichuan University, Chengdu, Sichuan, China; 2 Department of Neurology, Jianyang People’s Hospital, Chengdu, Sichuan, China

**Keywords:** ALS-plus syndrome, amyotrophic lateral sclerosis, *CACNA1A* gene, CAG repeats, SCA, spinocerebellar ataxia

## Abstract

Amyotrophic lateral sclerosis (ALS), the most common type of motor neuron disease, primarily manifests as progressive weakness, atrophy, fasciculations, bulbar palsy, and pyramidal tract symptoms. Accumulating evidence indicates that the pathological spectrum of ALS extends beyond the pyramidal and neuromuscular motor systems, involving additional brain regions, manifesting as ALS-plus syndrome. We present a case of an elderly woman with bulbar-onset ALS accompanied by cerebellar manifestations and an intermediate-length *CACNA1A* allele. Based on the Gold Coast criteria, ALS diagnosis was made. Notably, the patient exhibited cognitive impairment and a positive Romberg sign, suggesting a broader phenotypic spectrum. Genetic analysis showed a CAG repeat genotype of 10/20 in the *CACNA1A* gene. The patient’s son carried a 14/20 genotype and displayed isolated cerebellar ataxia without motor neuron features. We reviewed the literature on spinocerebellar ataxia (SCA) co-occurring with motor neuron disease and discussed the uncertain significance of the intermediate-length *CACNA1A* allele in this context, weighing coincidental co-occurrence against a potential causal link. To our knowledge, this case is the first reported instance of an intermediate-length *CACNA1A* allele co-occurring with ALS in Chinese population, although the association between the allele and ALS remains unclear.

## Case report

**FIGURE 1:**
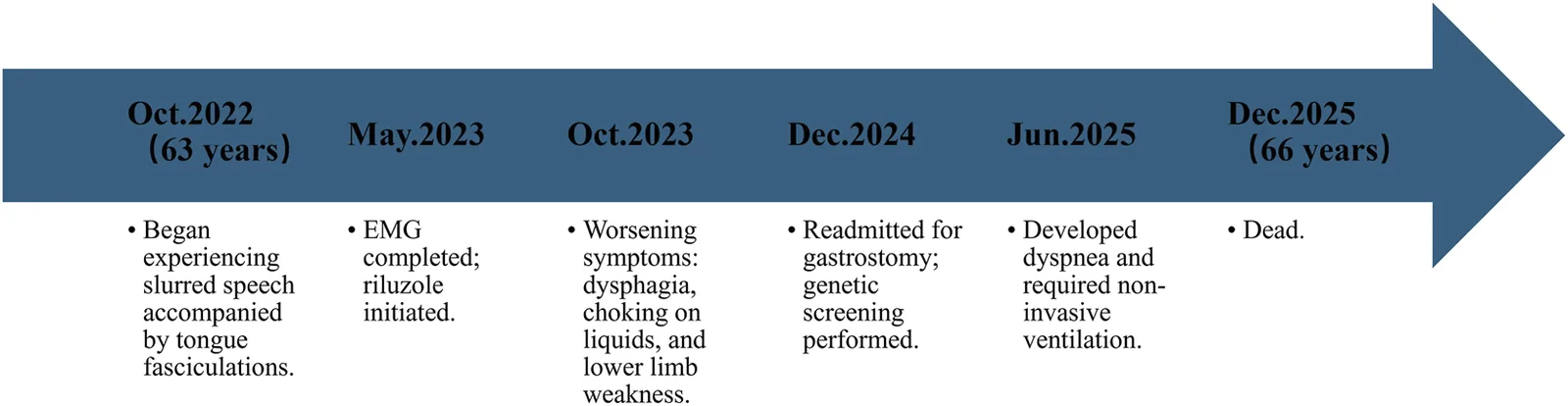
The timeline of the disease.

We described the case of a 66-year-old female. A timeline illustrating the clinical course and major disease-related events is presented in Figure 1. She began experiencing slurred speech accompanied by tongue fasciculations at the age of 63. Seven months later, as her condition progressed, she became increasingly anxious and visited our hospital for further evaluation. The blood test only showed mild anemia, while the electromyogram (Supplementary Table S1; Supplementary Tables S2, S3) revealed weakened F-wave responses in the bilateral median nerves. Acute and chronic denervation manifestations were observed in the bilateral anterior tibial muscles, the first interosseous muscles, the sternocleidomastoid muscles, and the T12 paravertebral muscles. However, the sensory nerve conduction was normal. The Amyotrophic Lateral Sclerosis Functional Rating Scale–Revised (ALSFRS-R) score was 42 (out of 48), the Hamilton Depression Rating Scale (HAMD) score was 4, the Hamilton Anxiety Rating Scale (HAMA) score was 6, the Activities of Daily Living (ADL) score was 100, the Frontal Assessment Battery (FAB) score was 13, and the Montreal Cognitive Assessment (MoCA) score was 17. A diagnosis of ALS was considered, and the patient was started on riluzole 50 mg twice daily, supplemented by multidisciplinary care (including respiratory care, swallowing management, and emotional support) (Andersen et al., 2012). Edaravone was not initiated because the patient was outside the population in which clinical trials have demonstrated the greatest likelihood of benefit. We monitored this ALS patient with follow-up assessments. One year after symptom onset, she began having trouble swallowing, coughing while drinking, and lower limb weakness. At this time, her ALSFRS-R score was 39. Twenty-six months after symptom onset, the patient’s dysarthria and limb weakness worsened. Eating had become increasingly difficult for her, and she was admitted to our hospital for gastrostomy placement.

Neurological examination revealed dysarthria, tongue muscle atrophy with fasciculations, weakness in the tongue and masseter muscles, drooling, and limb muscle atrophy (most pronounced in the interosseous muscles of both hands). Muscle tone was slightly increased, muscle strength was Medical Research Council (MRC) grade 4+, and tendon reflexes were hyperactive. Palmar and jaw reflexes were positive, with a positive finger-to-nose test and a Romberg sign, while the patient could not perform the tandem gait test. ALSFRS-R score was 32; Scale for the Assessment and Rating of Ataxia (SARA) was 17; International Cooperative Ataxia Rating Scale (ICARS) was 35.

The patient presented with progressive motor dysfunction and concurrent upper and lower motor neuron involvement. According to the Gold Coast criteria, a definite diagnosis of ALS could be established in this patient (Shen et al., 2021); however, it was unusual that this patient also presented with cerebellar ataxia symptoms. According to the literature, approximately 13.6% of ALS patients may have extrapyramidal symptoms, cerebellar impairment, or autonomic dysfunction (McCluskey et al., 2014).

Therefore, the following clinical features support consideration of an ALS-plus phenotype in this patient:

First, the patient presented with bulbar onset. Studies have shown that nearly half of ALS-plus syndrome patients present with bulbar onset, whereas this occurs in only 23.2% of non-ALS-plus patients (McCluskey et al., 2014).

Second, cognitive impairment was observed in our patient, with executive dysfunction being the predominant manifestation. We carefully examined the subscale scores of her cognitive assessments and found that her FAB impairment was primarily in executive function (inability to perform conflicting instructions), while her MoCA impairment was mainly in delayed recall, abstraction (word similarity), and visuospatial and executive abilities (clock drawing). The decreased MoCA score may be partly attributable to her educational level (9 years of schooling); however, the decreased FAB score and impaired executive function on the MoCA suggest frontotemporal dysfunction. Classical ALS has traditionally been regarded as representing motor symptoms caused by upper and lower motor neuron damage, without significant cognitive impairment. However, accumulating evidence indicates that the pathological spectrum of ALS extends beyond the pyramidal and neuromuscular motor systems, involving additional brain regions. Studies have shown that the incidence of pseudobulbar affect (PBA) and ALS-frontotemporal dementia (ALS-FTD)-related cognitive impairment in ALS-plus syndrome patients is more than twice that in classic ALS. Therefore, the impaired cognitive performance reflected by the decreased MoCA and FAB scores further supported the consideration of an ALS-plus phenotype in this patient.

Finally, there were cerebellar imaging abnormalities and clinical signs of cerebellar involvement. Brain MRI in this patient indicated cerebellar atrophy, and neurological examination revealed positive finger-to-nose test and Romberg sign. Although a positive Romberg sign may also result from large-fiber sensory or proprioceptive dysfunction, the patient’s nerve conduction results showed no sensory nerve conduction abnormalities; thus, cerebellar involvement was considered a more likely contributor to this finding. Moreover, cerebellar signs are recognized features of ALS-plus syndrome (McCluskey et al., 2014).

Therefore, from a clinical perspective, we considered an ALS-plus phenotype as a possible explanation for the multisystem involvement observed in this patient. Importantly, the observed phenotype could also represent ALS-plus with an incidental intermediate-length *CACNA1A* allele; therefore, the contribution of *CACNA1A* should be interpreted cautiously.

**FIGURE 2:**
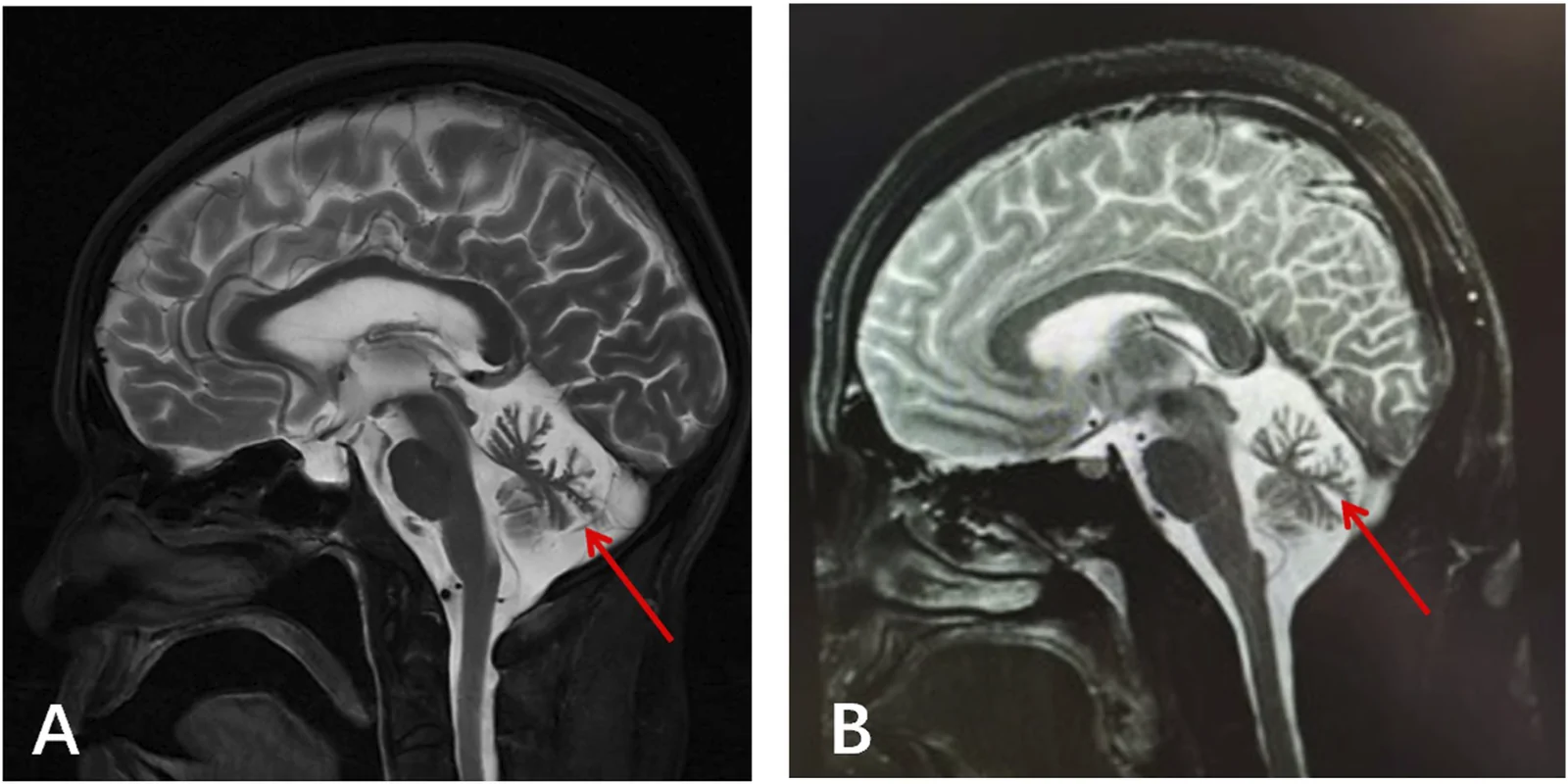
Head MRI of the patient and her son. Note: **(A)** In December 2024, an MRI scan of the patient indicated cerebellar atrophy. **(B)** An MRI of her son showed that the bilateral cerebral sulci were widened, and the bilateral cerebellar crus were narrowed.

**FIGURE 3:**
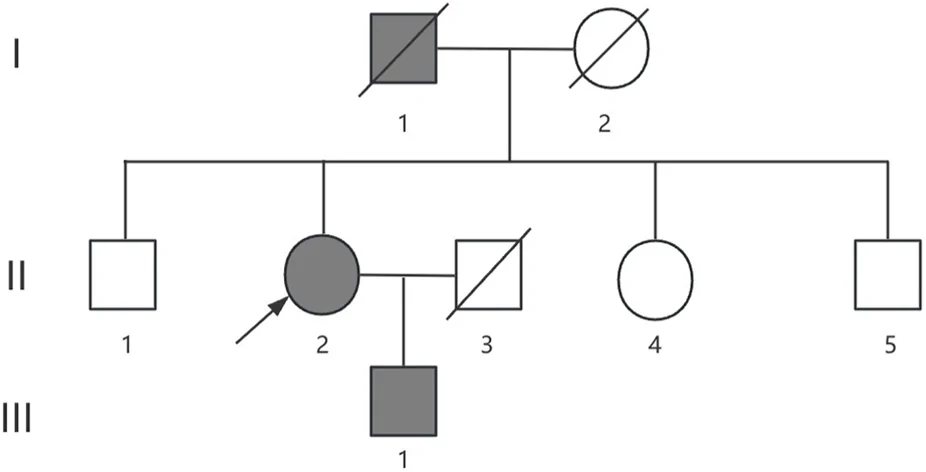
Family pedigree of the patient. Note: The square represents males; the circle represents females. Hollow symbols indicate non-affected members, while solid symbols indicate affected members. The underlined symbols represent deceased individuals. The arrows indicate the proband. I1: Passed away at the age of 75; cause of death unknown; symptoms of unsteady gait were present in the years preceding death. II2: Proband; other siblings have no contact. III1: 43 years old; had symptoms of slurred speech and unsteady gait.

To clarify the diagnosis, we performed a head MRI (Figure 2A), which showed cerebellar atrophy. We conducted whole-exome sequencing (WES) using the NGS method. Bioinformatics analysis of the WES data employed three repeat-aware algorithms, including TREDparse, ExpansionHunter, and GangSTR, to screen for repeat expansions in genes including *ATXN2*. No pathogenic *ATXN2* CAG repeat expansion was identified. Separately, targeted dynamic mutation analysis using fluorescent PCR and capillary electrophoresis revealed a *CACNA1A* CAG repeat genotype of 10/20. We also investigated the patient’s family history (Figure 3) and noted that her deceased father had symptoms of unsteady gait several years before his death. Additionally, her son showed deficits in the finger-to-nose test, rapid alternating movements, and tandem walking. His SARA score was 9, and ICARS score was 20. Brain MRI of her son (Figure 2B) showed bilateral widening of the sulci and atrophy of the superior cerebellar peduncles. His genetic analysis revealed a CAG repeat number of 14/20 in the *CACNA1A* gene.

The patient exhibited clinical features suggestive of an ALS-plus phenotype and was found to carry an intermediate-length *CACNA1A* allele. At the 32-month follow-up from symptom onset, the patient was bedridden and required non-invasive ventilation due to respiratory distress. The patient died 38 months after the onset of symptoms.

## Review

We searched the literature in PubMed, China National Knowledge Infrastructure (CNKI) and Wanfang Data for an association between SCA and motor neuron disease/ALS and found only 18 such cases, including the present one (Table 1) (Ohara et al., 2000; Nanetti et al., 2009; Infante et al., 2004; Furtado et al., 2004; B et al., 2011; Ferrari et al., 2024; Jaques et al., 2021; Kim et al., 2013; Ono et al., 2025; Pedroso et al., 2016; Pinto and De Carvalho, 2008; Singh et al., 2017; Spataro and La Bella, 2014; Takubo et al., 2025; Tazen et al., 2013; Brenner et al., 2019). Among these, Ferrari et al. reviewed reported cases of spinocerebellar ataxia associated with motor neuron disease and highlighted the clinical and genetic heterogeneity of this overlap phenotype. Notably, *CACNA1A*-related cases represent only a small proportion of reported SCA–motor neuron disease overlap cases, and the causal relationship remains uncertain. Among these, 9 cases (50%) had SCA2, 4 cases (22.2%) had SCA3, 3 cases (16.7%) had SCA6, and there was one case each of SCA1 and SCA8. Of these, 11 (61.1%) were male, and 7 (38.9%) were female. Half of the patients first presented with cerebellar ataxia, followed by the development of muscle weakness or atrophy after variable intervals (the timeline was not clearly specified in some cases). Three patients (16.7%) also developed Parkinsonism, including limb rigidity and bradykinesia.

**TABLE 1:** Cases of SCA with motor neuron disease.

Case	Author	Report year	Sex	Age	Age at SCA onset	Age at MND onset	Main SCA symptoms	Main MND symptoms	Fasciculation	Tendon reflex	Dysarthrosis	Cerebellar atrophy	Electromyography	Family history	Final diagnosis	DNA analysis
1	Shinji Oharart et al.	1999	F	57	57	58	Progressive gait staggering, poor finger-to-nose test and heel-knee test, wide gait base	Muscular atrophy and muscle weakness; at the age of 64, required continuous ventilator support	(+) lingualis	The left ankle reflex has disappeared	(+)	(+)	Fibrillation potentials appeared in the tongue muscles and the muscles of the upper and lower limbs	(+) the father has ataxic gait	SCA6	14/24 repetitions of CAG
2	Sarah Furtado et al.	2004	M	70+	ND	ND	The eye movements are adequate, but the tracking is of the scanning type	Myasthenia and muscle atrophy	(+) diffuse	Normal	(+)	(−)	Compliant with ALS	(+) Nephew with ALS	SCA2	22/33 repetitions of CAG
3	Jon Infante et al.	2004	F	61	61	62	Postural imbalance, intermittent dysphagia	Myasthenia and muscle atrophy	(+)	ND	(+)	(+)	The muscles of the upper and lower limbs showed many fibrillation and fasciculation potentials	(+)	SCA2	35/22 repetitions of CAG
4	Susana Pinto et al.	2008	M	64	61	61	Gait ataxia	Progressive gait disorder, spasticity of both upper and lower limbs	(+) upper and lower limb muscles	Hyperactivity	(+)	(−)	The muscles of the limbs show widespread muscle fibrillation	(−)	SCA3	69 repetitions of CAG
5	Lorenzo Nanetti et al.	2009	F	50	50	66	Unsteady gait; at the age of 65, slow scanning movements, mild limb and gait ataxia occurred	Myasthenia and muscle atrophy	(+) Face, upper limbs	Weaken	(+)	(+)	Diffuse fibrillation and fascicular twitch potentials	(+) uncle has Parkinson’s disease	SCA2	39 repetitions of CAG
6	Pedro Braga-Neto et al.	2011	M	46	45	46	Slow scanning movement, mild limb and gait ataxia	Myasthenia and muscle atrophy	(+)	Hyperactivity	(+)	(+)	Muscle fasciculation, fibrillation and positive sharp wave potentials	(+) there were affected individuals (ataxia) in the first and second generations	SCA2 presents as ataxia-parkinsonism-motor neuron disease syndrome	40 repetitions of CAG
7	Sirinan Tazen et al.	2013	F	47	36	ND	Difficulty in walking, slow scanning eye movement, swaying of the trunk, and poetic-like speech	(−)	(+) Face, shoulders	Weaken	(+)	ND	The genial muscles exhibit muscle bundle tremors and muscle fiber fibrillation	(+) uncle has SCA2	SCA2	40/22 repetitions of CAG
8	Sirinan Tazen et al.	2013	M	62	ND	62	(−)	Difficulty in walking, muscle atrophy of the limbs, and increased muscle tone	(+) limbs	Hyperactivity	(+)	(−)	Diffuse loss of nerve innervation	(+) the niece has SCA2	ALS accompanied by complete CAG repeat expansion of ATXN2	39/22 repetitions of CAG
9	Ji Sun Kim et al.	2013	M	65	ND	63	Unsteady gait, vertical nystagmus	Difficulty in swallowing, generalized muscle atrophy	(+) Masticatory muscles, thigh muscles	Hyperactivity	(+)	(+)	Widespread loss of nerve innervation	(+) Brother has been diagnosed with SCA8	SCA8 proposed phenotype (ALS-like)	86 repetitions of CTA/CTG
10	Synofzik M et al.	2014	M	47	ND	ND	(−)	Myasthenia and muscle atrophy	(+)	Hyperactivity	(−)	(−)	Diffuse multiphasic motor unit potentials, fibrillation potentials and positive sharp waves	(+) SCA1 family	ALS (ATXN1 intermediate repeats)	33/33 repetitions of CAG
11	José Luiz Pedroso et al.	2016	M	53	33	ND	Unsteady gait, spontaneous nystagmus	Obvious distal muscle atrophy of both upper and lower limbs	(+) Masticatory muscle	Disappearance	(+)	ND	ND	ND	SCA3	23/78 repetitions of CAG
12	Furtado LV et al.	2017	M	59	49	55	Balance disorder	Pseudobulbar palsy, quadriplegia, bilateral ptosis of the eyelids, muscle atrophy	(+)	Hyperactivity	(+)	(+)	Compliant with ALS	(+) Brother has SCA2	SCA2-ALS	22/37 repetitions of CAG
13	David Brenner et al.	2018	F	ND	ND	ND	(−)	Muscle weakness and signs of upper and lower motor neuron involvement	ND	ND	(+)	(−)	Acute and chronic denervation in the cervical segment, and chronic denervation in the thoracic and lumbar segments	(+) father has SCA6	SCA6-ALS	8/24 repetitions of CAG
14	Cristina Saade et al.	2021	M	66	58	65	Progressive ataxia	Myasthenia and muscle atrophy	(+) Diffuse	Disappearance	ND	(+)	Diffuse loss of nerve innervation	(+)	SCA3	The ATXN3 gene has undergone 66 times of allele amplification and duplication
15	Valerio Ferrari et al.	2024	M	58	38	ND	Progressive postural instability, gait ataxia, and slowed scanning movements	Numbness in the limbs, decreased muscle tone in the limbs, atrophy of the biceps muscles on both sides	(+)	Disappearance	(+)	(+)	A large amount of progressive denervation activity	(+) the father, the uncle and the cousin all have SCA2	SCA2-ALS	38 repetitions of CAG
16	Shohei Ono et al.	2025	F	53	25	51	Unsteady gait. At the age of 40, she developed ataxic dysarthria, slow eye movement while scanning, and wide base of gait	Progressive upper limb weakness and muscle atrophy, as well as atrophy of the tongue muscles	(+) Masticatory muscle	Hyperactivity	(+)	(+)	The trapezius muscle and the biceps brachii muscle have active denervation of the nerve supply	The daughter of great-aunt has ALS	ALS associated with SCA2 and ATXN2 amplification	39/22 repetitions of CAG
17	Masato Takubo et al.	2025	M	62	40	61	Ataxic gait, mild ataxic dysarthria	Progressive weakness and atrophy of the right upper limb	(+) the trunk and limbs, as well as the tongue muscles	Hyperactivity	(+)	(+)	Diffuse muscle fasciculation potentials in the cervical spinal cord segments, acute and chronic denervation, as well as chronic denervation in the thoracic and lumbar spinal cord segments	(+) several relatives have been diagnosed with SCA3	SCA3 associated with sporadic ALS	73/19 repetitions of CAG
18	Xueping Chen et al.	2026	F	66	65	63	Positive finger-to-nose test, positive Romberg sign, and inability to perform tandem walking	Atrophy of the tongue muscles and limb muscles accompanied by tremors	(+)	Hyperactivity	(+)	(+)	Extensive neuronal damage	(+) son has an intermediate-length CACNA1A allele	ALS-Plus with an intermediate-length CACNA1A allele	10/20 repetitions of CAG

Abbreviations: ALS, amyotrophic lateral sclerosis; SCA, spinocerebellar ataxia; ND, no description.

## Discussion

ALS is the most common type of motor neuron disease, which is mainly manifested in progressive skeletal muscle weakness, atrophy, fasciculations, bulbar palsy and pyramidal tract signs (Meyer, 2021).

This case adds to these series of diseases, and based on the above review, to our knowledge, this is the first reported case of an intermediate-length *CACNA1A* allele co-occurring with ALS in the Chinese population. In other countries, the first case was described by Shinji Ohara et al. in Japan, involving a 57-year-old female (Ohara et al., 2000). In contrast to our case, this Japanese patient initially presented with cerebellar symptoms, including dysarthria and progressive gait ataxia, with muscle atrophy and fasciculations appearing 1 year later. Over the course of her illness, her limb weakness rapidly progressed, and by 7 years after onset, she was paralyzed and dependent on ventilator support. Electromyography indicated neuronal damage, head MRI revealed cerebellar cortical atrophy, and genetic analysis showed 14/24 CAG repeats. The second case was described by David Brenner et al., in 2018, involving a German female (age not specified) (Brenner et al., 2019). The clinical phenotype of this patient was unusual, as she presented with ALS symptoms without cerebellar ataxia, and her head MRI did not show cerebellar atrophy. However, genetic analysis revealed an 8/22 CAG repeat genotype.

Compared with the two cases analyzed above, this patient carried only an intermediate-length *CACNA1A* allele. Although a definitive diagnosis of SCA6 could not be established, her son also harbored the same allele and exhibited cerebellar ataxia manifestations, including scanning speech, gait instability, and cerebellar atrophy, suggesting that the intermediate-length *CACNA1A* allele may be associated with cerebellar vulnerability in this family, although its clinical significance remains uncertain. However, this uncertainty complicates the diagnostic interpretation. Below, we discuss several possible explanations.

The first possibility is coincidental co-occurrence of the two conditions. We considered two scenarios: (1) A patient with ALS-Plus syndrome coincidentally carrying an intermediate-length *CACNA1A* allele; and (2) sporadic ALS occurring in a family segregating the intermediate *CACNA1A* allele.

The second possibility is a potential biological relationship between the two conditions. We speculate that even if not a definitive causative variant, it may represent one component within a broader neurodegenerative susceptibility framework. Meanwhile, we acknowledge that any potential role of the intermediate-length *CACNA1A* allele in ALS remains speculative and requires further investigation.

From a clinical-genetic perspective, the observation that the son carries the same intermediate-length allele and cerebellar phenotype but does not exhibit ALS-related manifestations argues against a direct causal role of *CACNA1A* in motor neuron degeneration.

Previous studies have shown that *CACNA1A* gene CAG repeat expansions are neither a common cause of sporadic ALS nor familial ALS (Brenner et al., 2019; Lee et al., 2011). Any potential role of *CACNA1A* CAG repeat expansion in motor neuron degeneration remains hypothetical, particularly for intermediate-length alleles such as the 20-repeat variant identified in this patient. Unlike the intermediate-length *ATXN2* CAG repeat expansions, which are an established risk factor for ALS (Matsumura and Futamura, 2001), the molecular mechanisms by which *CACNA1A* CAG repeat expansion might relate to motor neuron degeneration remain unclear.

The *CACNA1A* gene encodes the α1 subunit of the CaV2.1 channel (also known as the P/Q-type calcium channel) complex, which forms the ion-conducting pore. The CaV2.1 channel is a voltage-gated Ca^2+^ channel primarily located on the presynaptic membrane of the brain. Pathogenic variants in the *CACNA1A* gene, including CAG repeat expansions and point mutations, cause a spectrum of neurological disorders collectively termed CaV2.1 channelopathies (Ma et al., 2025). Pathogenic *CACNA1A* expansions, particularly those associated with SCA6, have been proposed to disrupt calcium homeostasis and neuronal function. Abnormal calcium signaling has been proposed to contribute to mitochondrial dysfunction, oxidative stress, and neuronal vulnerability. Importantly, disturbances in calcium homeostasis, mitochondrial impairment, oxidative stress, and excitotoxicity are also recognized mechanisms involved in ALS pathogenesis (Van Den Bosch et al., 2006; King et al., 2016). Excessive intracellular calcium accumulation may increase metabolic stress and render motor neurons, which have a high metabolic demand and extensive axonal projections, particularly susceptible to degeneration. Therefore, *CACNA1A*-mediated calcium dysregulation represents a biologically plausible but currently unproven mechanism linking CaV2.1 dysfunction to neuronal vulnerability. However, whether an intermediate-length 20-repeat *CACNA1A* allele, as observed in our patient, is sufficient to induce similar functional alterations remains unknown. At present, the contribution of this intermediate allele to motor neuron vulnerability remains speculative. However, patients with *CACNA1A* gene mutations often present symptoms of episodic ataxia type 2 (Ophoff et al., 1996), familial hemiplegic migraine (Ophoff et al., 1996), and spinocerebellar ataxia type 6 (Zhuchenko et al., 1997). In contrast, cases involving motor neuron degeneration are extremely rare, and the mechanisms underlying the occurrence of motor neuron disease features in patients with *CACNA1A*-related disorders remain unclear.

In *ATXN2*, the normal CAG repeat range is approximately 22–23. Repeat numbers exceeding 34 cause SCA2, while intermediate-length repeat sequences (27-33) significantly increase the risk of ALS (Lee et al., 2011). Whether a similar threshold exists for *CACNA1A* in the context of ALS remains unknown. In current reports of *CACNA1A* gene combined with ALS, the CAG repeat numbers in patients were 14/24 and 8/22. Generally, the normal CAG repeat sequence in *CACNA1A* ranges from 4 to 18 repeats, with 19–20 repeats considered to be an intermediate range of uncertain significance (Hatano et al., 2025). A repeat range of more than 21 is considered pathogenic (Hatano et al., 2025), and typical SCA6 alleles ranging from 21 to 25 repeats (Ishikawa et al., 1997). In our case, both the mother and the son carried only 20 CAG repeats. Although a definitive diagnosis of SCA6 cannot be established based on the current literature, we cannot exclude the possibility that this intermediate-length *CACNA1A* allele may influence the clinical phenotype through an unknown mechanism; however, this hypothesis remains speculative and requires further investigation.

The patient’s son, who is also a carrier of the intermediate-length *CACNA1A* allele, provided the following perspective: “My mother initially developed slurred speech and unsteady gait. At first, we thought these symptoms were simply related to aging; however, her condition deteriorated rapidly. She became increasingly anxious and frustrated as she gradually lost the ability to eat and communicate clearly. The diagnosis was difficult for our family to understand—she was initially diagnosed with ALS, and later genetic testing identified the *CACNA1A* intermediate-length allele. We still do not know whether these findings are directly related. She experienced significant suffering during the final months of her illness, and we hope that sharing her case may help clinicians recognize similar complex presentations earlier.”

This case report has several limitations. First, the patient’s MMSE score was unavailable, and neither PET imaging nor formal neuropsychological testing was performed, precluding a comprehensive evaluation of her cognitive status. Nevertheless, the decreased MoCA and FAB scores are consistent with executive dysfunction. Second, the *CACNA1A* 20-repeat allele falls within the intermediate range of uncertain significance; without dose-dependent support (e.g., homozygosity or a larger expansion), its pathogenicity remains unclear. Third, repeat expansion analysis was performed using WES-derived data. ATXN2 CAG repeat expansions were assessed, whereas C9orf72 repeat expansion could not be definitively excluded due to the limitations of WES-based detection. Therefore, the contribution of a pathogenic *C9orf72* repeat expansion to the clinical phenotype cannot be completely ruled out. Fourth, the patient’s son harbored the same allele and presents with an isolated cerebellar phenotype; based on current information, he shows no ALS-related symptoms. This appears to argue against the association between the *CACNA1A* allele and ALS. Finally, the father’s genetic status could not be confirmed.

This case highlights the importance of evaluating cerebellar signs in patients with ALS. Such non-motor features add to the clinical complexity of ALS and underscores the importance of comprehensive genetic evaluation and cautious interpretation of borderline variants in patients with complex phenotypes. Further studies are required to determine whether intermediate-length *CACNA1A* alleles represent incidental findings or modifiers of complex neurodegenerative phenotypes.

## Data Availability

The original contributions presented in the study are included in the article/Supplementary Material, further inquiries can be directed to the corresponding author.
