# Supplementary material for: Amyotrophic lateral sclerosis -plus patient with an intermediate-length CACNA1A allele: a Case Report
Source: Front Genet. 2026 Aug 19;17:1860828. doi: 10.3389/fgene.2026.1860828 (PMC13534756; doi:10.3389/fgene.2026.1860828)
Supplement: Supplementary file 1 [file Supplementaryfile1.docx]

**Table S1: Electromyography of the patient-** **Motor Nerve Conduction**

| MOTOR NERVES: | Latency (SD)  (ms) | Amplitude (SD)  (mV) | CV (SD)  (m/s) | Amp % (SD)  (%) | F (SD)  (ms) |
| --- | --- | --- | --- | --- | --- |
| Right Medianus |  |  |  |  | 24.3 |
| Wrist - APB | 2.8 | 13.2 | N/A | N/A |  |
| Bel Elb - Wrist | 6.5 | 12.1 | 51.4 | -8 |  |
| Left Medianus |  |  |  |  | 23.3 |
| Wrist-APB | 2.7 | 9.9 | N/A | N/A |  |
| Bel Elb-Wrist | 6.0 | 9.5 | 54.5 | -1 |  |
| Right Ulnaris |  |  |  |  | 23.7 |
| Wrist-ADM | 2.1 | 11.4 | N/A | N/A |  |
| Bel Elb-Wrist | 5.9 | 11.8 | 55.3 | 3 |  |
| Left Ulnaris |  |  |  |  | 23.8 |
| Wrist-ADM | 2.2 | 11.7 | N/A | N/A |  |
| Bel Elb-Wrist | 5.5 | 10.9 | 56.1 | -7 |  |
| Right Tibialis |  |  |  |  | 42.7 |
| Ande-AHB | 3.7 | 11.2 | N/A | N/A |  |
| Left Tibialis |  |  |  |  | 44.8 |
| Ande-AHB | 4.1 | 12.8 | N/A | N/A |  |
| Right Peroneus |  |  |  |  | 49.0 |
| Ankle-EDB | 3.4 | 3.4 | N/A | N/A |  |
| Be knee-Ankle | 9.9 | 2.7 | 45.4 | -19 |  |
| Left Peroneus |  |  |  |  | 50.5 |
| Ankle-EDB | 4.3 | 3.2 | N/A | N/A |  |
| Be knee-Ankle | 9.9 | 3.6 | 46.4 | 15 |  |

**Note:** The F-wave occurrence rate of the bilateral median nerves is low. CV: Conduction Velocity; Amp: Amplitude; APB: Abductor Pollicis Brevis; Bel: Biceps Brachii; ADM: Abductor Digiti Minimi (manus); Ande: Abductor Digiti Minimi (pedis); AHB: Abductor Hallucis Brevis; EDB: Extensor Digitorum Brevis; SD: Standard Deviation.

**Table S2: Electromyography of the patient- Sensory Nerve Conduction**

| SENSORY NERVES: | Latency (SD)  (ms) | Amplitude (SD)  (mV) | CV (SD)  (m/s) | Amp % (SD)  (%) |
| --- | --- | --- | --- | --- |
| Right Medianus |  |  |  |  |
| Dig III-Wrist | 1.96 (-2.1) | 27 | 58.7 | N/A |
| Left Medianus |  |  |  |  |
| Dig III-Wrist | 1.92 (+0.4) | 30 | 65.1 | N/A |
| Right Ulnaris |  |  |  |  |
| Dig V-Wrist | 1.67 (-2.9) | 22 | 59.9 | N/A |
| Right Ulnaris |  |  |  |  |
| Dig V-Wrist | 1.48 (-3.4) | 20 | 60.8 | N/A |
| Right Suralis |  |  |  |  |
| Ankle-Foreleg | 1.77 | 17 | 53.7 | N/A |
| Left Suralis |  |  |  |  |
| Ankle-Foreleg | 1.92 | 16 | 52.1 | N/A |

**Note:** Sensory nerve conduction studies of the median, ulnar, and sural nerves were normal bilaterally. CV: Conduction Velocity; Amp: Amplitude; Dig: Digitorum; SD: Standard Deviation.

**Table S3: Electromyography of the patient-** **Needle EMG**

|  |  | Amp | | | | Dur | | | | Area | | | | Poly |
| --- | --- | --- | --- | --- | --- | --- | --- | --- | --- | --- | --- | --- | --- | --- |
| Muscle | # | uA | reLSD | <min | >max | ms | reLSD | <min | >max | uAms | reLSD | <min | >max | (%) |
| Right Tibialis anterior | 10 | 548 | -0.1 | 0 | 0 | 11.6 | 0.1 | 0 | 0 | 1024 | N/A | N/A | N/A | 50 |
| Left Tibialis anterior | 13 | 1233 | 2.8 | 0 | 4 | 12.6 | 0.9 | 0 | 0 | 1827 | N/A | N/A | N/A | 31 |
| Right Interosseous | 12 | 743 | 0.5 | 0 | 0 | 11.2 | 1.3 | 0 | 0 | 1088 | N/A | N/A | N/A | 42 |
| Left Interosseous I | 6 | 1702 | 3.3 | 0 | 0 | 11.9 | 1.8 | 0 | 0 | 2741 | N/A | N/A | N/A | 0 |
| Left Gastrocnemius caput mediale | 15 | 765 | N/A | N/A | N/A | 11.2 | N/A | N/A | N/A | 1229 | N/A | N/A | N/A | 0 |
| Right Gastrocnemius caput mediale | 12 | 704 | N/A | N/A | N/A | 11.8 | N/A | N/A | N/A | 1239 | N/A | N/A | N/A | 17 |
| Right APB | 17 | 1043 | N/A | N/A | N/A | 12.1 | N/A | N/A | N/A | 1813 | N/A | N/A | N/A | 6 |
| Left Sternocleidomastoid | 19 | 1590 | N/A | N/A | N/A | 13.8 | N/A | N/A | N/A | 2387 | N/A | N/A | N/A | 11 |
| Left Paravertebral T12 | 14 | 861 | N/A | N/A | N/A | 11.0 | N/A | N/A | N/A | 1431 | N/A | N/A | N/A | 14 |

**Note:** Acute and chronic denervation manifestations were observed in the bilateral anterior tibial muscles, the first interosseous muscles, the sternocleidomastoid muscles, and the paraspinal muscles at T12 level. Amp: Amplitude; APB: Abductor Pollicis Brevis; Dur: Duration; reLSD: Relative Latency Side Difference.
